# A pilot test of the Adult Sickle Cell Quality of Life Measurement Information System (ASCQ-Me) and the Jenerette Self-Care Assessment (J-SAT) Tools in adults with sickle cell disease

**DOI:** 10.1186/s40814-019-0471-0

**Published:** 2019-07-04

**Authors:** Dominique Bulgin, Christian Douglas, Paula Tanabe

**Affiliations:** Duke University School of Nursing, Durham, USA

**Keywords:** Health-related quality of life, Patient-reported outcomes, PROMIS, ASCQ-Me, Sickle cell disease

## Abstract

**Background:**

The purpose of this study was to pilot test two sickle cell-specific instruments, the Adult Sickle Cell Quality of Life Measurement Information System (ASCQ-Me) and Jenerette Self-Care Assessment Tool (J-SAT), to determine recruitment rate, percent completion of the instrument battery, and patient perceptions of health-related quality of life outcomes and self-care activities in a convenience sample of adults with sickle cell disease (SCD).

**Methods:**

A cross-sectional pilot study was conducted. Participants were recruited from a sickle cell clinic and conference on SCD. Subjects completed self-administered assessments including demographic and clinical characteristics, ASCQ-Me, and the J-SAT.

**Results:**

Twenty of 22 participants completed the instruments (2 refusals) and most instruments had 100% completion rates. Participants reported average to healthier status on ASCQ-Me measures than a normative referent population of 556 individuals with SCD. Participants also reported high disease severity and high J-SAT scores (mean = 30.2), indicating frequent participation in self-care activities.

**Conclusions:**

There was good participation, low refusal rates, and subjects completed the instruments and items without difficulty. Based on this work, a multi-method, multi-site study in Jamaica and the USA will be conducted to understand the relationships between health-related quality of life, stigma, and self-management in adults with SCD.

## Background

Sickle cell disease (SCD) is the most common genetically inherited hemoglobin disorder and occurs in 1 out of every 365 African American births [1]. Individuals with SCD suffer from a variety of serious health complications including renal failure, vaso-occlusive crisis, leg ulcers, acute*,* and chronic pain [2]. Complications are associated with significantly shortened lifespans for individuals with SCD, 42 for males and 48 for females in the US*A* [3]. In addition to increasing mortality, complications of SCD can negatively influence health-related quality of life (QoL), which has been found to be very low in this population [4–7]. McClish and Penberthy [7] found that individuals with SCD have worse QoL than the general population, and their QoL levels are most similar to individuals undergoing hemodialysis. For this paper, QoL is defined as health-related QoL. Health-related quality of life refers to the impact of health status on quality of life [5].

QoL in SCD can be affected by how well individuals manage their disease. Complications of SCD affect all organ systems. Thus self-management of sickle cell disease is complex, including tasks such as hydration, sleep, blood transfusions, frequent visits with hematologist and other specialists, and managing multiple medications, including opioids [2]. In order to effectively self-manage SCD, individuals need to participate in self-care activities, which Jenerette *and* Murdaugh 2008 describe as “engaging in therapeutic activities and actively accessing resources to maintain or improve health status and quality of life.” [8] Limited exploration of SCD self-management and self-care activities has been conducted [9, 10]*,* despite the integral role these concepts have in influencing the QoL of those affected by SCD.

To understand the complexity of the relationships between self-care activities and QoL, reliable and valid measures must be utilized to study these concepts. SCD*-*specific measures have been developed for both QoL and self-care activities. The Adult Sickle Cell Quality of Life Measurement Information System (ASCQ-Me) was developed using the same methodology as the Patient-Reported Outcome Measurement Information System (PROMIS). The SCD*-*specific measures of the ASCQ-Me can be used to complement the more general content of the PROMIS measures. The ASCQ-Me consists of seven subscales: *e*motional *i*mpact, *s*leep *i*mpact, *s*ocial *f*unctioning *i*mpact, *s*tiffness *i*mpact, *p*ain *i*mpact, and *p*ain *e*pisode *f*requency and *s*everity that were determined to be the most salient QoL indicators amongst individuals with SCD [11–13]. The ASCQ-Me’s SCD specific items assessing physical, mental, and social health can be used in conjunction with or separately from PROMIS measures. The Jenerette Self-Care Assessment Tool (J-SAT) was developed to measure the frequency that individuals with SCD participate in self-care activities [8].

Studies assessing the reliability and validity of the ASCQ-Me have been conducted, while studies detailing the development of J-SAT are unpublished. A brief search of PubMed for the terms “ASCQ-Me” and “Adult Sickle Cell Quality of Life Measurement Information System” revealed that only a few studies have been published on the ASCQ-Me and the majority report on the psychometrics and development of the ASCQ-Me [11–13]. The J-SAT has been utilized in descriptive, cross-sectional studies [8, 10]. In order to begin to better understand QoL and self-management in SCD, it is important to pilot test these instruments in a sample of individuals with SCD. The objective of this research is to generate information that is useful to those interested in using the ASCQ-Me and J-SAT to assess QoL and self-care activities in adults with SCD. The purpose of this pilot study is to test the ASCQ-Me and J-SAT instruments to determine *(*1) recruitment rate, *(*2) percent completion of the instrument battery, and *(*3) patient perceptions of QoL outcomes and self-care activities in a convenience sample of adults with SCD. Results of this study will inform the development of a multi-method, multi-site study in Jamaica and the *USA* to understand the relationships between health-related quality of life, stigma*,* and self-management in adults with SCD.

## Methods

### Design

A cross-sectional survey design was used. Institutional review board approval was obtained from Duke University and patients provided informed written consent prior to participation.

### Setting

Subjects were recruited from the following two sources: (1) an annual local SCD conference, and (2) an adult comprehensive SCD clinic in North Carolina. The conference is 2 days, held in North Carolina, and approximately 100 individuals with SCD attend. Subjects were also recruited from an adult comprehensive SCD clinic which provides SCD care and health maintenance for patients ages 18 and older. The clinic has approximately 700 patients on record, approximately 2500 visits a year, with 400 unique patient visits.

### Sample

Inclusion criteria were > 18 years of age, ability to read English, and self-reported diagnosis of one of the following SCD genotypes: HbSS , HbSC, Hb SB0, or SB+.

### Recruitment and procedures

Subjects were recruited during the SCD conference and in clinic. The study was announced to approximately 100 attendees several times during the conference, and interested individuals were instructed to approach the study investigator. Subjects recruited at clinic were first approached by a healthcare provider who assessed their interest in participating in the study and then introduced them to a research coordinator if interested. Twelve individuals were approached in clinic. After obtaining informed consent, subjects were given a battery of measures to complete. Surveys were completed using paper and pencil. All participants were compensated for their time in the form of a $10 gift card.

### Measures

#### Demographics

Participants completed a demographics questionnaire (age, gender, race, ethnicity, education, employment, children, relationship status, insurance, income, and SCD genotype).

#### Disease severity

The ASCQ-Me Medical History Checklist (SCD-MHC) is a 9-item survey that lists the most common treatments and conditions associated with SCD, such as avascular necrosis, kidney disease, and blood transfusions. The scale uses a dichotomous yes/no response system to indicate presence of the treatment or condition. The SCD-MHC is scored as the sum of endorsed questions. Higher scores indicate higher disease severity. Scores range from 0 to 9. During psychometric testing, SCD-MHC was significantly related to age, healthcare utilization, and vaso-occlusive pain episodes; but not to comorbidity questions which referred to conditions not associated with SCD [12]. The SCD-MHC is the most common instrument used to assess disease severity in SCD.

#### Quality of life (ASCQ-Me)

SCD is highly complex and is associated with high disease severity; thus, a generic QoL measure such as PROMIS is not always sufficient to determine QoL [11]. The ASCQ-Me was rigorously developed to measure health-related QoL specifically for individuals living with SCD. Development of ASCQ-Me included a comprehensive literature review of patient-reported outcomes, a series of interviews with patients with SCD and SCD healthcare providers, and item bank development using a field test population of 556 individuals with SCD recruited from 7 clinics located across the USA [12, 13]. The field test respondents had sociodemographic characteristics that were consistent with characteristics of adults who have SCD [11]. ASCQ-Me consists of 5-item banks with 5 corresponding short forms (to assess emotional, pain, sleep, social functioning, and stiffness impact) and another 5-item fixed form to assess pain episode frequency and severity. Pain episodes are fixed form that provides separate scores for pain episode frequency (2 items) and severity (3 items). Raw scores range 0–11 for frequency and 0–22 for severity. To make the raw scores comparable, a *z* score is obtained and then a *t* score transformation is performed. Higher scores indicate worse health status for pain episode frequency and severity.

The short forms are scored on Likert scales ranging 1–5 from “never” to “always” or “not at all” to “very much.” Raw scores range from 5 to 25 and response-pattern scores, which are based on item-response theory and expressed as logits, can range from − 4 to + 4. Response-pattern scores undergo a *t* score transformation to have a mean of 50 and standard deviation of 10 indicates an average health score on the scale. ASCQ-Me uses a normative referent population based on the responses of 556 adults with SCD that were surveyed during the development of the instrument. Individuals in the referent population were majority female (64%), varied in sickle cell genotype (majority had hemoglobin SS followed by hemoglobin SC), and ranged in age from 18 to 65 and above. [14]. The value of 50 indicates the health score of the average field test respondent during testing of the ASCQ-Me, and the value of 10 represents one standard deviation unit. Higher scores indicate healthier status on the subscale. Internal consistency reliability was between 0.80 and 0.73 for the fixed form and 0.94 to 0.90 for short forms [11].

#### Self-care activities (J-SAT)

The J-SAT consists of eight items that measure self-care activities, defined as participating in activities and accessing resources that improve and maintain health and QoL. A Likert scale ranging from “never” to “almost always” is used and items include statements such as “I understand (know why I am taking) my medications” and “I avoid stress whenever possible.” The J-SAT had an internal consistency reliability of .80 during the development of the instrument. Scores range 4–32, with higher scores indicating greater frequency of self-care activities [8].

Across all measures, there were a total of 47 questions (9 disease severity, 30 ASCQ-Me, and 8 J-SAT items).

### Data analysis

There is only one way to score the pain episode frequency and severity fixed form. The pain episode subscale must be scored after obtaining separate raw scores for pain episode frequency and severity. *z* scores should be calculated for the ASCQ-Me measure raw scores. Next, a popular transformation or “*T*-score transformation” using the mean and standard deviation of the referent population should be performed on the *z* scores.

There are two methods to score the ASCQ-Me short forms (emotional, pain, sleep, social functioning, and stiffness impact). (1) The first method involves using the raw to *t* score conversion tables in the ASCQ-Me User’s Manual available on healthmeasures.net to score the subscales. (2) The second method uses the FREE HealthMeasures Scoring Service, provided on healthmeasures.net that calculates the item response theory pattern. An excel template is provided, as well as item banks and specific variable names that should be utilized.

For the current study, paper surveys were entered into REDCap. Descriptive statistics were obtained using SAS (Version 9.4) and the FREE HealthMeasures Scoring Service. We used both of the scoring methods described above. For the ASCQ-Me short forms, we compared results using the *t* score conversion table and FREE HealthMeasures Scoring Service methods; there was minimal discrepancy. While indeterminable, we attributed these minimal discrepancies to round-off error due to the approximation of *t*-scores in the conversion tables. As described in the ASCQ-Me User Manual, if only one item was missing from an ASCQ-Me measure the raw score was approximated by obtaining the sum of the four responses answered, multiplying by five which is the number of items on the short form, and then dividing by the number of items answered. ASCQ-Me short forms cannot be scored by hand if 3 or fewer (out of 5) items are answered [14].

## Results

**Fig. 1:**
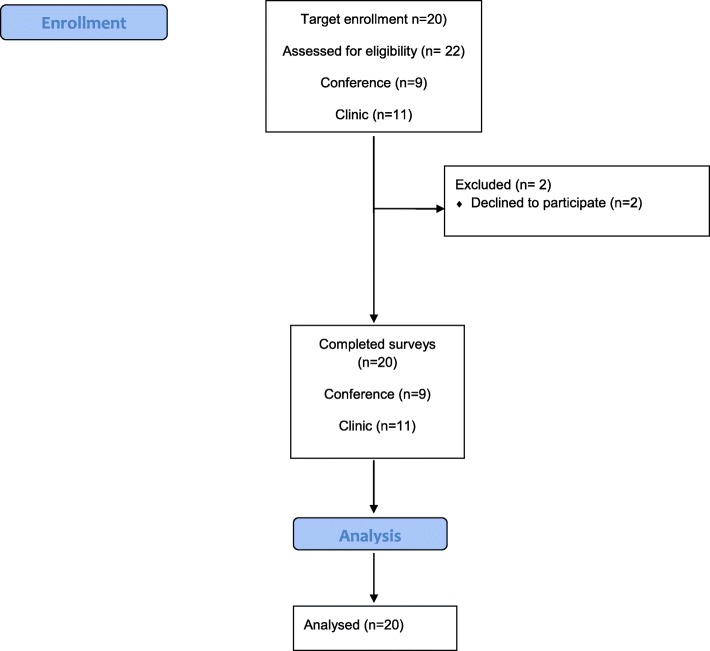
CONSORT diagram

Twenty-two individuals were assessed for eligibility (conference *n* = 10, clinic *n* = 12). Two individuals refused participation (1 in clinic and 1 at the conference) when the study was explained and informed consent sought, resulting in a total of 20 participants (Fig. 1). Sixty percent reported being female; and all except one reported being Black or African American. Mean age was 38.5 ± 13.7 and ranged from 22 to 67 (see Table 1).

**Table 1:** Demographics

Characteristic	Total sample (*n* = 20), *n* (%)
Age	38.5 ± 13.7
18–24	2.0 (10.0)
25–34	7.0 (35.0)
35–44	5.0 (25.0)
45–54	3.0 (15.0)
55–64	2.0 (10.0)
65–74	1.0 (5.0)
Sex	
Male	8.0 (40.0)
Female	12.0 (60.0)
Race	
Black or African American	19.0 (100.0)
Ethnicity	
Not-Hispanic/Latino	18.0 (100.0)
Social status	
Single	15.0 (75.0)
Married	4 .0 (20.0)
Divorced	1.0 (5.0)
Children	
0	13.0 (65.0)
1	3.0 (15.0)
2	3.0 (15.0)
3	1.0 (5.0)
Education	
High school/GED	2.0 (10.0)
Some college	5.0 (25.0)
Trade or vocational training	3.0 (15.0)
Bachelor’s degree	5.0 (25.0)
Graduate school	5.0 (25.0)
Yearly income	
Less than $11,000	7.0 (35.0)
$20,001–$30,000	2.0 (10.0)
$30,001–$40,000	3.0 (15.0)
$50,001–$75,000	3.0 (15.0)
$75,001–$100,000	3.0 (15.0)
More than $100,000	2.0 (10.0)
Genotype	
HbSS	10.0 (52.63)
HbSC	8 .0 (42.11)
Hb SS/a-thalassemia	1 .0 (5.26)

On average, participants completed the battery of surveys in 15 min. The following surveys had a 100% completion rate: emotional impact, pain episode frequency and severity, sleep impact, social functioning impact, stiffness impact, and the J-SAT. The pain impact scale had a 95% completion rate (1 participant missed 1 item), and the ASCQ-Me SCD-MHC had a 90% completion rate (2 participants missed 1 item on the eye damage and pain medication SCD-MHC questions respectively). Participants were able to complete the survey without assistance. Table 2 reports results from the ASCQ-Me measures and J-SAT. Briefly, subjects reported high disease severity with average or healthier status on the ASCQ-Me measures than the referent population and frequent participation in self-care activities.

**Table 2:** ASCQ-Me (*t* scores*) and J-SAT results

		Minimum	Maximum	Mean	SD	SE
ASCQ-Me SCD-MHC**	*n* (%)	4.0	7.0	5.6	1.1	-
Low disease severity	3 (15)					
Medium disease severity	3 (15)					
High disease severity	14 (70)					
ASCQ-Me quality of life						
Emotional impact		37.9	65.5	53.7	8.7	1.9
Pain impact		38.1	63.8	50.6	7.7	1.7
Social functioning impact		43.5	69.8	53.7	9.6	2.1
Stiffness impact		35.5	65.4	52.4	8.5	1.9
Sleep impact		35.0	61.1	50.2	8.4	1.9
Pain frequency		25.0	56.3	43.6	9.9	2.2
Pain severity		21.8	59.3	42.5	10.1	2.3
J-SAT		27.0	32.0	30.2	1.8	0.4

*SD* = standard deviation; *SE* = standard error

*The Adult Sickle Cell Quality of Life Measurement Information System uses a *t*-score metric, calibrated with a referent population. Fifty is the mean, and 10 is the standard deviation of the reference population. A mean of 50 indicates average health status on the subscale

**Scores on the SCD Medical History Checklist range 0–9 and are obtained by summing the number of endorsed responses. Low (< 2), medium (= 2), and high (> 2) disease severity are cut-offs and are based on specifications by Keller, Yang, Tredwell, and Hassell 2017

## Discussion

Considering the chronicity of SCD and the complexity of SCD self-management, understanding more about QoL for these individuals is essential to improving health status in SCD. To the best of our knowledge, we report the first use of ASCQ-Me besides the original psychometric work. Overall, the use of the ASCQ-Me is feasible, completion rates were 100% for all subscales except the SCD-MHC and pain impact. Using paper and pencil, participants were able to quickly complete the instruments with low burden and patient refusal rate was very low.

Overall, participant scores indicated that they had high disease severity, but average or heathier status on the ASCQ-Me measures than normative scores in the referent population. Pain and sleep impact was consistent with the referent population, meaning that participants reported average health status on these measures. Participants had slightly healthier status than the referent population for stiffness, social functioning, and emotional impact. Participants in this sample reported being almost one standard deviation healthier than average on pain episodes subscale, indicating less frequent and severe pain episodes.

Our sample reported high disease severity. In the testing and comparison of ASCQ-Me to PROMIS, participant SCD-MHC scores were split into low (< 2), medium (= 2), and high (> 2) categories; the cut-offs for the scores were based on tertiles of the distribution of the score in the reference population [11]. Seventy percent of participants in our sample had high disease severity (mean = 5.6, SD = 1.1). Despite the majority of our sample having high disease severity, the scores on the ASCQ-Me measures suggest that our participants had an average or healthier QoL than the referent population. Due to small sample sizes, we were unable to conduct analyses which might have resulted in a better understanding of the observed relationships between the SCD-MHC and the ASCQ-Me short-form scores. Individuals with medium and high disease severity in the referent population (*n* = 556) had worse health status than our sample for all ASCQ-me measures (emotional impact 49.75 and 47.04; pain impact 48.88 and 46.30; social functioning impact 48.91 and 46.72; stiffness impact 48.81 and 45.81; sleep impact 48.79 and 48.10; pain frequency 50.33 and 51.99; pain severity 50.33 and 52.10 respectively) [11].

Our sample (*n* = 20) had a similar race, sex, age, and SCD genotype (> 50% = HbSS) breakdown to the referent population. While our sample was recruited from a SCD clinic and conference, respondents in the referent population were recruited all across the USA using diverse methods including clinics, flyers, online, and word of mouth [12]. Our sample was highly educated; however, the education level was not described for the referent population. It is important to note that in the testing of ASCQ-Me and the comparison of ASCQ-Me to PROMIS, adults with SCD were always less healthy than the general population, even those with low disease severity. Previous studies testing PROMIS in those with high SCD disease severity indicated worse health compared to the general population, with magnitude differences ranging 0.5 to 1.1 standard deviation units [11]. Thus, designation of average and healthier status in our population does not necessarily indicate good or better health than others, but average health and healthier status when compared to the referent population of adults with SCD. Health status on the QoL indicators assessed in the ASCQ-Me may be poorer in our population, if compared to general populations. Individuals with SCD have been found to have poor QoL [4–7], even more so for those with increased disease severity [15] and higher pain frequency and severity [7]. Using a sickle cell-specific QoL instrument could vastly improve our understanding of QoL for individuals with SCD.

Participants in our sample also had high J-SAT scores (mean = 30.2), indicating frequent participation in activities that improve health and QoL. The healthier status of our pilot sample is expected considering that our sample has access to a comprehensive SCD care setting and frequently participates in self-care activities. Furthermore, the population we surveyed is highly educated, 90% have some college or higher educational attainment. In a 2015 study, Matthie, Jenerette, and McMillian found that in a sample of 103 young adults with SCD, self-care was significantly related to social support, SCD self-efficacy, and years of education [10]. Further research in a larger sample size is needed to determine if participation in self-care activities can mitigate the effect of disease severity on QoL for individuals with SCD. Despite the limited generalizability of these findings, the knowledge gained about the use and scoring of ASCQ-Me is essential for future research using these instruments to measure QoL in adults with SCD. We found it very feasible to administer ASCQ-Me in a clinic and public setting.

The scoring service provided by HealthMeasures is easy to use, but we learned several lessons that may be helpful when using this service. Becoming familiar with the scoring services prior to collecting data and developing a database is crucial. It is important to use these resources when developing databases, codebooks, and data dictionaries. We were not aware of these resources and developed our own variable names and field attributes that had to be changed later as they were not recognized by the scoring services. HealthMeasures provides a Microsoft Excel Scoring Service Input Template that asks for a PIN or participant identifier, the number of the assessment being scored, the item identification or variable names, and the item response score. The participant identifier and assessment number can be assigned by the individual seeking to score the ASCQ-Me short form(s). Multiple assessments, or ASCQ-Me short forms can be entered into one Microsoft Excel Scoring Service Input Template so it is important to number each assessment individually. For instance, all social functioning forms can be assigned an assessment number of 1, and all stiffness short forms can be assigned 2. The variable names and item response scores must be consistent with what is assigned by HealthMeasures.net. Missing responses must be left blank because if only one item is missing from a subscale the score is approximated by the scoring services using item response theory. Finally, to minimize data entry error, we suggest administering the surveys on computers, smartphones, or tablets. The Microsoft Excel Scoring Service Input Template and FREE HealthMeasures Scoring Services can be obtained at https://www.assessmentcenter.net/ac_scoringservice, while the ASCQ-Me forms, including variable names and item response scores, are available at http://www.healthmeasures.net/index.php?option=com_instruments&view=search&Itemid=977.

### Limitations

The primary limitation of this study is the small sample size and use of a convenience sample which limits generalizability. Our sample was too small to make statistical comparisons; thus, results of this study cannot accurately depict true differences between this sample of 20 individuals and the referent population of 556 surveyed during the development of the ASCQ-Me. However, one of the purposes of this project was to evaluate the recruitment rate and the ability of subjects to complete the assessment tools. There is also a limitation to note in the scoring of the SCD-MHC, wherein higher scores indicate higher disease severity. Each item is not an equal representation of disease severity; it is possible that two individuals could score the same and one have higher or lower disease severity than the other.

## Conclusions

It was possible to administer the ASCQ-Me and J-SAT in a clinic and public setting with relative ease and in a short period of time. Both of these instruments can help improve our understanding of QoL for individuals with SCD. The 20 individuals surveyed in this pilot study reported: (1) high disease severity, (2) average or healthier status on the ASCQ-Me measures than the normative referent population, and (3) very frequent participation in self-care activities. Our team used this pilot data to conduct a multi-method, multi-site study in Jamaica and the USA to understand the relationships between health-related quality of life, stigma, and self-management in adults with SCD. This pilot project was critical to the success of the larger study.

## Data Availability

The datasets generated and/or analyzed during the current study are not publicly available to protect the privacy of the individuals involved in this study but are available from the corresponding author on reasonable request.
